# Fulminant Acute Chest Syndrome and Multiorgan Failure in Sickle Cell Disease With Chronic Hepatic Vasculopathy: A Fatal Synergy

**DOI:** 10.7759/cureus.103987

**Published:** 2026-02-20

**Authors:** Sofia Prada, Raquel Silva, Ana Sílvia Coelho, Marinela Major, Paulo Freitas

**Affiliations:** 1 Department of Internal Medicine, Unidade Local de Saúde de Amadora/Sintra, Lisbon, PRT; 2 Faculty of Medicine, Universidade de Lisboa, Lisbon, PRT; 3 Department of Critical Care Medicine, Unidade Local de Saúde de Amadora/Sintra, Lisbon, PRT

**Keywords:** acute chest syndrome, hepatic vaso-occlusive disease, hyperhemolysis, multiorgan failure, plasma exchange, sickle cell disease, sickle hepatopathy

## Abstract

Sickle cell disease (SCD) is a multisystem disorder characterized by recurrent vaso-occlusive events and progressive organ damage. Among its chronic complications, sickle hepatopathy, driven by sinusoidal obstruction, ischemia, and cholestasis, remains underrecognized despite its major prognostic significance. During acute crises, hepatic dysfunction may amplify systemic inflammation, impair metabolic reserve, and precipitate rapid multiorgan failure. We report the case of a 28-year-old man with homozygous SCD and biopsy-confirmed chronic hepatic vaso-occlusive disease who presented after discontinuation of hydroxyurea with severe hemolysis, profound hyperbilirubinemia (total bilirubin 59 mg/dL), and anemia. Shortly after admission, he developed acute hypoxemic respiratory failure with new bilateral pulmonary infiltrates consistent with acute chest syndrome (ACS). Despite aggressive supportive management, including red blood cell transfusion, corticosteroids, and plasma exchange (PLEX) for worsening hemolysis and coagulopathy, his condition rapidly deteriorated, with progressive metabolic acidosis, disseminated intravascular coagulation, and hepatorenal failure. Exchange transfusion was deemed unsafe due to critically low hemoglobin (2.3 g/dL), and both extracorporeal membrane oxygenation and urgent liver transplantation were contraindicated. The patient died less than 24 hours after admission to the ICU. This case highlights the fulminant and often fatal interaction between advanced sickle hepatopathy and ACS. Chronic hepatic dysfunction compromises cytokine clearance, exacerbates endothelial injury, and reduces physiological resilience, thereby increasing vulnerability to hyperhemolysis and multiorgan failure. While PLEX has been proposed as a rescue therapy in severe hyperhemolysis or hepatic vaso-occlusive crisis, evidence remains limited, and outcomes are poor once profound acidosis and systemic collapse ensue. In patients with SCD and preexisting hepatic disease, the development of ACS should prompt immediate multidisciplinary escalation and early consideration of exchange transfusion before irreversible organ dysfunction occurs. This case underscores that sickle hepatopathy is a dynamic, high-risk condition that critically influences outcomes during acute SCD crises.

## Introduction

Sickle cell disease (SCD) is a complex inherited hemoglobinopathy caused by a single nucleotide substitution in the β-globin gene (HBB), resulting in the production of abnormal hemoglobin S (HbS) [1]. Under conditions of deoxygenation, HbS polymerizes, leading to erythrocyte deformation, membrane damage, and increased cellular adhesion, which together drive chronic hemolysis and recurrent vaso-occlusive events [1]. SCD follows an autosomal recessive inheritance pattern, with homozygous HbSS disease representing the most severe phenotype. The condition predominantly affects individuals of African, Mediterranean, Middle Eastern, and Indian ancestry, reflecting the historical protective advantage of the sickle trait against malaria [1].

Worldwide, SCD affects more than five million individuals, with the highest prevalence observed in sub-Saharan Africa, although migration patterns have led to increasing global distribution, including in Europe and North America. Advances in neonatal screening, infection prevention, and disease-modifying therapies have significantly improved survival into adulthood; however, this has been accompanied by a growing burden of chronic organ dysfunction and long-term complications [2]. Among these complications, hepatic involvement, commonly referred to as sickle hepatopathy, remains an important yet underrecognized contributor to morbidity [3,4]. Repeated episodes of sinusoidal obstruction, ischemia-reperfusion injury, and cholestatic dysfunction progressively impair hepatic metabolic capacity and immune homeostasis, predisposing patients to decompensation during acute vaso-occlusive events.

Acute chest syndrome (ACS) remains the leading cause of hospitalization and mortality in patients with SCD [5]. Its pathophysiology is multifactorial and may involve infection, fat embolism, transfusion-related hemolysis, pulmonary vaso-occlusion, or a combination of these mechanisms [6]. Notably, the coexistence of ACS and chronic hepatic dysfunction amplifies systemic inflammation, oxidative stress, and hypoxemia, thereby creating a high-risk clinical phenotype for rapid progression to multiorgan failure [4].

We report a fatal case of fulminant ACS in a patient with homozygous SCD (HbSS) and underlying chronic hepatic vaso-occlusive disease who developed advanced multiorgan failure despite early intensive management. This case underscores the devastating interplay between hepatic vaso-occlusion, severe hemolysis, and pulmonary injury in the advanced stages of sickle hepatopathy.

## Case presentation

A 28-year-old man living in Portugal with HbSS, congenital asplenia, and biopsy-proven chronic hepatic vaso-occlusive disease presented to the ED with a three-day history of progressive fatigue, generalized weakness, and right thigh pain. He had discontinued hydroxyurea (2,000 mg/day) three weeks prior to presentation. His baseline hemoglobin ranged between 7.5 and 8.5 g/dL, with chronic hyperbilirubinemia (total bilirubin 13-16 mg/dL; direct bilirubin 9-11 mg/dL), consistent with stable sickle hepatopathy.

On arrival, the patient was afebrile, tachypneic (respiratory rate 26 breaths/min), and markedly icteric, but hemodynamically stable. Initial laboratory evaluation (Table 1) revealed severe anemia (hemoglobin 4.3 g/dL), reticulocytosis (10.8%), marked leukocytosis (19.9 × 10⁹/L), thrombocytopenia (78 × 10⁹/L), mild coagulopathy (INR 1.4), and acute kidney injury (creatinine 1.7 mg/dL). Liver function tests demonstrated profound hyperbilirubinemia (total bilirubin 59 mg/dL; direct bilirubin 48 mg/dL) with a cholestatic pattern (alkaline phosphatase 930 U/L, gamma-glutamyl transferase 355 U/L). Elevated lactate dehydrogenase (1,189 U/L) and undetectable haptoglobin confirmed ongoing hemolysis.

**Table 1:** Laboratory tests This table details serial laboratory findings obtained during hospitalization, illustrating the progression of biochemical and hematological abnormalities over time. ALP, alkaline phosphatase; ALT, alanine aminotransferase; aPTT, activated partial thromboplastin time; AST, aspartate aminotransferase; GGT, gamma-glutamyl transferase; HbS, hemoglobin S; INR, international normalized ratio; LDH, lactate dehydrogenase; PCT, procalcitonin; PLT, platelets; PLEX, plasma exchange; SOFA, sequential organ failure assessment; TP, prothrombin time

Parameter (units)	Reference range	Admission	5 hours post-admission	13 hours post-admission	24 hours post-admission	32 hours post-admission	35 hours post-admission
Hemoglobin (g/dL)	13-17	4.3	3.9	5	5.6	2.3	5.3
HbS (%)	%	-	-	-	49.1	44.6	20.5
Reticulocytes (%/abs)	0.5-2.5/50-100 × 10⁹/L	10.8/159 × 10⁹/L	-	-	26.3/492 × 10⁹/L	-	-
Leukocytes (×10⁹/L)	4-10	19.9	20	18.9	33.3	46	41.5
Platelets (×10⁹/L)	150-410	78	85	83	76	57	81
D-dimer (ug/L)	<500	-	-	-	903	-	-
Fibrinogen (g/L)	1.8-3.5	-	-	-	5.4	2	1.9
aPTT (seconds)	23-32	62.1	-	65.2	56.6	>160	>160
INR	<1.2	1.4	-	1.4	1.3	1.5	1.5
TP (seconds)	10-12	17	-	17	14	17	18
Haptoglobin (mg/dl)	30-200	-	<10	-	-	-	-
AST (U/L)	<40	229	-	225	238	143	202
ALT (U/L)	<41	55	-	51	59	35	43
ALP (U/L)	40-130	930	-	872	823	-	-
GGT (U/L)	<60	355	-	316	297	-	-
LDH (U/L)	135-225	1189	-	-	1168	-	-
Total bilirubin (mg/dL)	<1.2	59.3	-	54.9	61.2	35.5	40.3
Direct bilirubin (mg/dL)	<0.2	48.3	-	47.1	51.7	26.6	33.9
Creatinine (mg/dL)	0.7-1.2	1.7	2.1	1.8	1.9	2.5	3.9
Urea (mg/dL)	<50	76	81.5	91.9	97.1	100.6	102.9
Na⁺ (mmol/L)	136-145	130.9	130.6	132.8	132.9	147.6	151.7
K⁺ (mmol/L)	3.5-5.1	2.74	3.24	3.89	3.1	5.3	5.15
Ca²⁺ (mg/dL)	8.6-10.0	-	-	8.3	8.4	7.3	7.6
Troponin (ng/L)	<14	6.6	-	-	-	-	-
CRP (mg/dl)	<0.50	5.1	4.8	4.3	4.4	-	-
PCT (ng/ml)	<0.05	-	10.5	-	-	-	-
SOFA score		7			11		14
Comments					ICU admission	After PLEX	

The patient received packed red blood cell transfusions early during hospitalization. Despite transfusion support, hemoglobin levels subsequently declined below baseline values, with worsening hyperbilirubinemia and persistent markers of hemolysis, raising concern for hyperhemolysis syndrome. A direct antiglobulin test was performed and was negative, and no new alloantibodies were identified. There was no prior history of delayed hemolytic transfusion reactions. The temporal relationship between transfusion exposure, worsening hemolysis, and subsequent clinical deterioration supported the diagnosis of hyperhemolysis.

**Figure 1:**
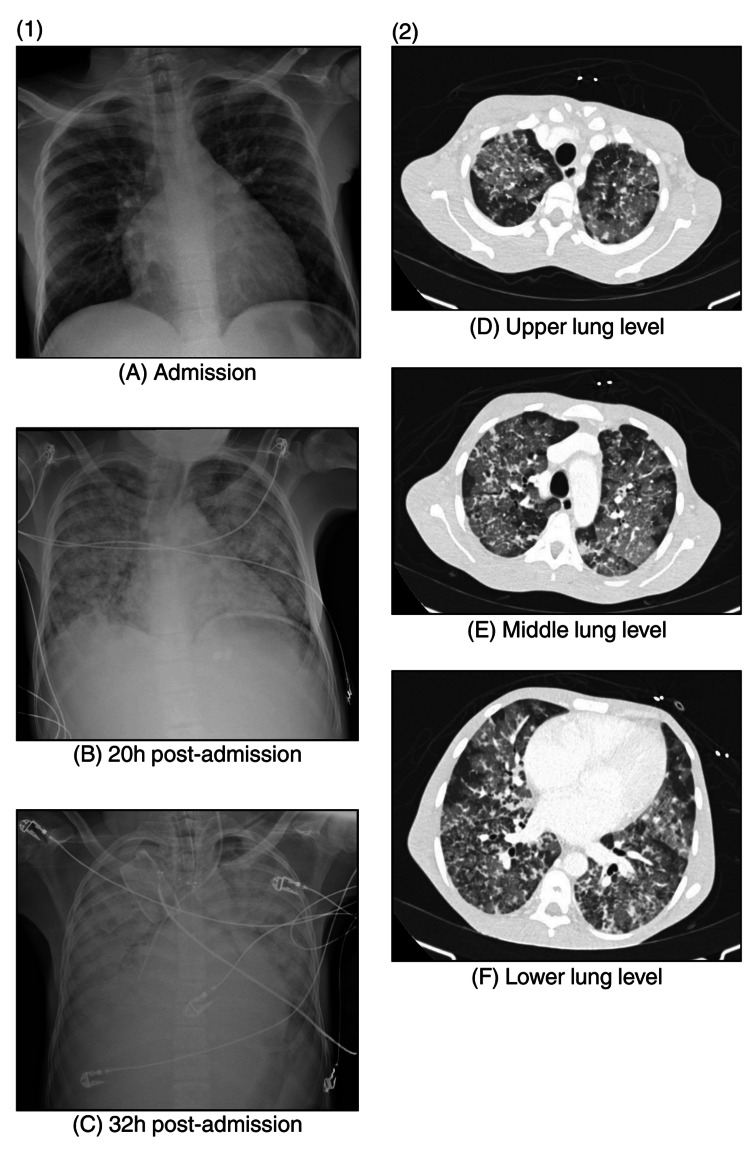
Progressive radiographic worsening of the chest showing new bilateral alveolar infiltrates (1) Sequential chest radiographs showing rapid progression from normal findings (A) to bilateral patchy infiltrates (B) and diffuse alveolar consolidation (C) within 30 hours, compatible with ACS. (2) High-resolution CT of the chest: axial images at the upper (D), middle (E), and lower (F) lung levels demonstrating diffuse bilateral ground-glass opacities with areas of confluent consolidation, a radiological pattern typical of ACS. ACS, acute chest syndrome

By late evening, oxygen saturation had declined to 78% on room air, accompanied by dyspnea, hemoptysis, and diaphoresis. Chest CT revealed bilateral ground-glass opacities and alveolar consolidation (Figure 1), findings consistent with ACS.

He was admitted to the ICU approximately 24 hours after hospital admission and initiated on high-flow nasal oxygen (FiO₂ 40-60%, flow 60 L/min), pulse-dose corticosteroids, broad-spectrum intravenous antibiotics, and plasma exchange (PLEX) (1.5 plasma volumes using 5% albumin and fresh frozen plasma in a 50:50 ratio). Following PLEX, the patient appeared clinically less tachypneic, with partial improvement in several biochemical parameters. Despite a marked decline in hemoglobin to 2.3 g/dL, total bilirubin (35.5 mg/dL), creatinine (2.5 mg/dL), fibrinogen (2.0 g/L), and platelet count (57 × 10⁹/L) all improved compared with pre-exchange values, suggesting transient stabilization of hemolysis and coagulopathy. Given the severity of anemia and concerns regarding procedural safety in the context of profound anemia and evolving metabolic instability, red blood cell exchange transfusion was considered inappropriate at that time, and a single simple transfusion was performed instead, increasing hemoglobin to 5.3 g/dL.

Despite apparent biochemical stabilization, serial arterial blood gas analyses (Table 2) demonstrated progressive metabolic deterioration, with worsening acidemia. Values declined from a near-compensated state on ED admission (pH 7.36, bicarbonate 18.6 mmol/L, lactate 1.79 mmol/L) to severe metabolic acidosis on ICU admission (pH 7.13, bicarbonate 8.3 mmol/L, lactate 11.5 mmol/L), and subsequently to profound acidemia (pH 6.79, bicarbonate 8.2 mmol/L, lactate 13.35 mmol/L), consistent with refractory tissue hypoxia and circulatory failure.

**Table 2:** Serial arterial blood gases This table details serial arterial blood gas analyses performed during hospitalization, illustrating the evolution of acid-base balance and gas exchange abnormalities over the patient’s clinical course. Hb, hemoglobin; HFNC, high-flow nasal cannula; PLEX, plasma exchange

Parameter (units)	Reference range	Admission	20 hours post-admission	24 hours post-admission	32 hours post-admission	35 hours post-admission
FiO₂ (%)		21	35	60 (HFNC)	100	100
pH	7.35-7.45	7.363	7.358	7.321	7.13	6.786
pCO₂ (mmHg)	35-45	33	31.4	34.9	25	56.6
pO₂ (mmHg)	70-100	114	51.5	82.7	76.9	56
HCO₃⁻ (mmol/L)	21-26	18.6	17.3	17.6	8.3	8.2
Lactate (mmol/L)	<1.8	1.79	2.73	2.1	11.5	13.35
SaO₂ (%)	>96	98	85.7	95.5	86.7	71.4
Hb (g/dL)	14-18	4.2	6.1	5.2	2.6	5.9
Comments			After the second RBC	ICU admission	After PLEX	

Refractory hypoxemia ensued, necessitating endotracheal intubation. Rapid sequence intubation was performed, during which old blood was aspirated through the endotracheal tube, raising suspicion of diffuse pulmonary hemorrhage. Despite maximal ventilatory and medical support, adequate oxygenation could not be restored. The liver transplant team determined that urgent transplantation was not indicated, and extracorporeal membrane oxygenation (ECMO) was declined due to severe acidosis and coagulopathy. The patient progressed rapidly to multiorgan failure and died within 24 hours of ICU admission.

## Discussion

This case illustrates the catastrophic interaction between chronic sickle hepatopathy, hyperhemolysis, and ACS, culminating in fulminant multiorgan failure. While ACS remains the leading cause of mortality in adults with SCD, outcomes are particularly poor when severe hepatic dysfunction is present, suggesting that preexisting organ injury substantially reduces physiological reserve during acute crises [5,6].

Sickle hepatopathy represents a heterogeneous spectrum of liver injury driven by sinusoidal obstruction, ischemia-reperfusion injury, iron overload, and chronic hemolysis [3,4]. Over time, progressive hepatic dysfunction impairs detoxification pathways, cytokine clearance, and coagulation homeostasis, thereby amplifying endothelial activation and systemic inflammation during vaso-occlusive events. In advanced stages, this creates a pro-inflammatory and pro-thrombotic milieu that may predispose patients to rapid clinical deterioration once acute complications occur.

In the present case, hyperhemolysis likely acted as a central pathophysiological trigger linking hepatic decompensation and pulmonary injury. The temporal association between transfusion exposure, worsening hemolysis, and respiratory deterioration is consistent with previously described hyperhemolysis-associated ACS mechanisms [7,8]. Massive intravascular hemolysis releases free hemoglobin and heme, which promote nitric oxide scavenging, oxidative stress, endothelial dysfunction, and microvascular occlusion, thereby contributing to pulmonary injury and multiorgan dysfunction [1].

Recent discontinuation of hydroxyurea may also have contributed to disease destabilization. Hydroxyurea increases fetal hemoglobin production, reduces HbS polymerization, and decreases erythrocyte adhesion and leukocyte activation. Withdrawal of therapy is associated with increased hemolytic activity and vaso-occlusive risk, which may have lowered the threshold for severe complications in this patient with preexisting hepatic disease [1,6].

PLEX is not a standard therapy for ACS but has been proposed as a rescue strategy in severe hyperhemolysis or hepatic vaso-occlusive crisis, with the theoretical benefit of removing circulating free hemoglobin, inflammatory mediators, and pathogenic antibodies [3,7,8]. In this case, PLEX was associated with transient biochemical improvement, suggesting partial modulation of the hemolytic and inflammatory cascade. However, the subsequent progression to severe metabolic acidosis and disseminated coagulopathy underscores that once systemic collapse develops, therapeutic interventions may have limited impact.

Red blood cell exchange transfusion is generally considered the cornerstone intervention for severe ACS, particularly in patients with rapid clinical deterioration [5,6]. In our patient, exchange transfusion was deferred because of critically low hemoglobin levels and rapidly evolving metabolic instability, raising concerns regarding procedural safety. This highlights an important clinical dilemma: the therapeutic window for exchange transfusion may be narrow in patients with advanced organ dysfunction, and delays related to clinical instability may further reduce the likelihood of reversibility.

Multidisciplinary evaluation also confirmed the limited role of advanced rescue therapies in this context. Severe metabolic acidosis and coagulopathy are recognized contraindications to ECMO, and advanced hepatic fibrosis precluded urgent liver transplantation. Reports of ECMO support in SCD remain scarce, with outcomes strongly dependent on early initiation before irreversible multiorgan failure develops [9,10].

Importantly, this case reinforces that chronic sickle hepatopathy should not be considered merely a comorbidity but rather a major prognostic determinant in adult SCD. Hepatic dysfunction appears to act as a disease amplifier during acute crises by increasing systemic inflammation, impairing metabolic reserve, and accelerating progression toward multiorgan failure.

From a clinical perspective, several lessons emerge. First, strict adherence to disease-modifying therapy such as hydroxyurea remains essential, particularly in patients with established organ damage. Second, early recognition of hepatic involvement may help identify patients at higher risk of severe complications. Third, rapid escalation and early consideration of exchange transfusion before the onset of profound metabolic derangement may represent the most critical modifiable factor influencing outcomes.

To our knowledge, reports describing the combined impact of advanced sickle hepatopathy, hyperhemolysis, and fulminant ACS remain limited, highlighting the importance of recognizing this high-risk clinical phenotype.

## Conclusions

ACS in patients with advanced sickle cell hepatopathy may follow a rapidly progressive and fatal course, as illustrated in this case. The combination of massive hemolysis, hepatic dysfunction, and pulmonary injury can create a self-amplifying pathophysiological cascade that precipitates multiorgan failure within a short timeframe, moving from compensated dysfunction to irreversible collapse despite intensive care management. This report highlights that chronic sickle hepatopathy should be recognized as a major prognostic determinant rather than a simple comorbidity in adult SCD. Preexisting hepatic dysfunction appears to significantly reduce physiological reserve and increase vulnerability to severe complications during acute crises. Strict adherence to disease-modifying therapy, particularly hydroxyurea, remains essential to reduce hemolytic burden and vaso-occlusive risk.

Early identification of clinical deterioration and timely consideration of exchange transfusion before the development of profound metabolic derangement may represent the most critical modifiable factor influencing outcomes in this high-risk population. Multidisciplinary coordination involving hematology, hepatology, critical care, and transplant teams is crucial, although therapeutic options may become limited once advanced organ failure is established. Recognition of this high-risk clinical phenotype may help improve risk stratification and guide earlier escalation of care in patients with SCD and hepatic involvement. Future studies are needed to better define optimal management strategies and therapeutic windows in patients with combined sickle hepatopathy and severe acute complications.
